# A simulation of the random and directed motion of dendritic cells in chemokine fields

**DOI:** 10.1371/journal.pcbi.1007295

**Published:** 2019-10-07

**Authors:** Avery Parr, Nicholas R. Anderson, Daniel A. Hammer

**Affiliations:** 1 Harriton High School, Rosemont, Pennsylvania, United States of America; 2 Department of Chemical and Biological Engineering, University of Pennsylvania, Philadelphia, Pennsylvania, United States of America; 3 Department of Bioengineering, University of Pennsylvania, Philadelphia, Pennsylvania, United States of America; Northeastern University, UNITED STATES

## Abstract

Dendritic cells (DCs) are the most effective professional antigen-presenting cell. They ferry antigen from the extremities to T cells and are essential for the initiation of an adaptive immune response. Despite interest in how DCs respond to chemical stimuli, there have been few attempts to model DC migration. In this paper, we simulate the motility of DCs by modeling the generation of forces by filopodia and a force balance on the cell. The direction of fliopodial extension is coupled to differential occupancy of cognate chemokine receptors across the cell. Our model simulates chemokinesis and chemotaxis in a variety of chemical and mechanical environments. Simulated DCs undergoing chemokinesis were measured to have a speed of 5.1 ± 0.07 μm·min^-1^ and a persistence time of 3.2 ± 0.46 min, consistent with experiment. Cells undergoing chemotaxis exhibited a stronger chemotactic response when exposed to lower average chemokine concentrations, also consistent with experiment. We predicted that when placed in two opposing gradients, cells will cluster in a line, which we call the “line of equistimulation;” this clustering has also been observed. We calculated the effect of varying gradient steepness on the line of equistimulation, with steeper gradients resulting in tighter clustering. Moreover, gradients are found to be most potent when cells are in a gradient of chemokine whose mean concentration is close to the binding of the K_d_ to the receptor, and least potent when the mean concentration is 0.1K_d_. Comparing our simulations to experiment, we can give a quantitative measure of the strength of certain chemokines relative to others. Assigning the signal of CCL19 binding CCR7 a baseline strength of 1, we found CCL21 binding CCR7 had a strength of 0.28, and CXCL12 binding CXCR4 had a strength of 0.30. These differences emerge despite both chemokines having virtually the same K_d_, suggesting a mechanism of signal amplification in DCs requiring further study.

## Author summary

Dendritic cells harvest and display antigen to other immune cells, and motility is essential to their function. Dendritic cells use filopodia to pull themselves forward, and orient their filopodia based on signals received from chemokines. We developed a model of dendritic cell motion based on a force balance, in which pulling from filopodia (calculated from a well-established model of filopodial adhesion-clutch dynamics) is counterbalanced by adhesive friction, and where the angular orientation of filopodia is based on the strength of chemotactic signal. The model can explain directional motion in chemotactic gradients, as well as the effect of superposition of coincident gradients of chemokine on chemotaxis. The model also identifies conditions in which dendritic cells can be focused at a single location when confronted with countergradients of chemokine. The model makes specific prediction regarding dendritic cell motion and defines conditions for the design of extracorporeal devices for focusing the position of dendritic cells.

## Introduction

The adaptive immune system relies on antigen-presenting cells to bring antigen from peripheral tissues to secondary lymphoid organs (SLOs). Dendritic cells (DCs) are the most effective antigen presenting cells and have become a subject of great interest in recent years [1–3]. Immature DCs migrate through peripheral tissues until exposure to inflammatory cytokines or recognition of pathogenic antigen causes maturation [4]. Once mature, DCs migrate quickly to SLOs and present antigen to T cells [5]. To fulfil their duties effectively, DCs must be able to both move quickly and exhibit directional motion in response to chemical signals. In mature DCs, the principal chemokine receptors are CXCR4 and CCR7 [6]. CXCR4 binds the chemokine CXCL12, while CCR7 can bind to either CCL19 and CCL21. CCL19 and CCL21 are both overexpressed in SLOs and are important for the recruitment of DCs [7], indicating that CCL19 and CCL21 guide DCs towards SLOs.

Directional motion is governed by receptor occupancy of chemokine receptors; differential occupancy leads to chemotaxis. In addition, cells in linear gradients exhibit a decreased chemotactic response when the average concentration of ligand is too high [7,8]. This suggests that cells do not directly sense differences in chemokine concentration, but differences in the fraction of occupied receptors, a consistent feature of receptor-mediated chemotaxis [9].

**Fig 1:**
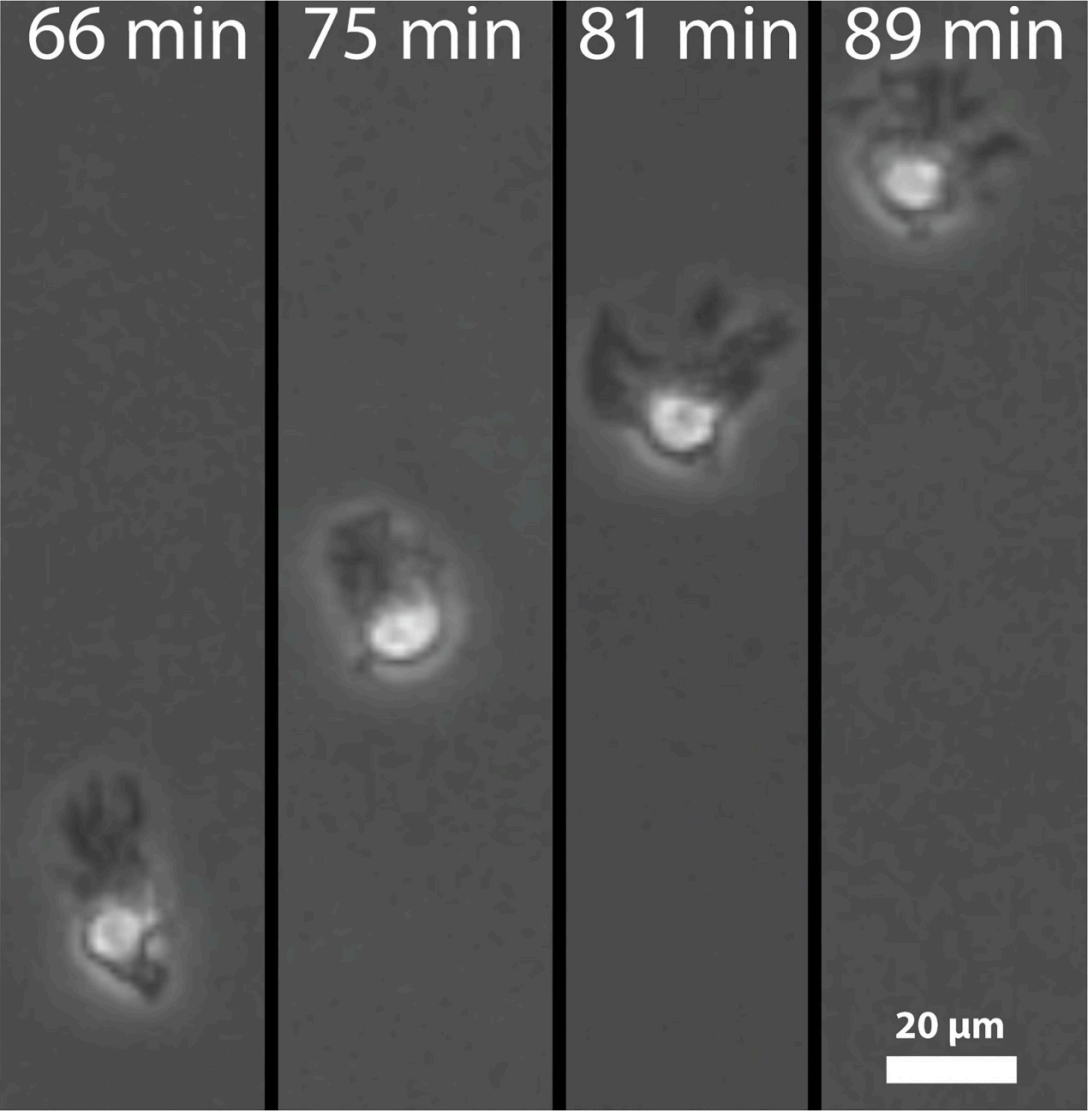
DCs crawl up chemokine gradients by focusing their filopodia along their direction of motion. Screenshots were taken from videos used for calculations by Ricart *et al*. [8].

Our lab has previously conducted experiments on the directional motion of dendritic cells in response to gradients of chemokine using microfluidic gradient chambers [8]. Under the gradient of a single chemokine, cells oriented so that their filopodia were directed toward the higher concentration of chemokine (Fig 1, adopted from video data reported in reference 7). When two gradients in opposing directions were used, cells preferentially migrated up one of the two gradients. Specifically, we exposed DCs to counter gradients of CCL19, CCL21, and CXCL12. Cells always preferred to migrate up gradients of CCL19, even in the presence of CCL21, which also signals through CCR7. However, cells showed little preference and migrated randomly when subjected to counter gradients of CCL21 and CXCL12. Gradients of CCL19 and a different chemokine required the slope of the CCL19 gradient to be up to 100 times shallower than the opposing gradient to cause random migration. In some cases, DCs migrated directionally in counter gradients to a central point, which we call the “line of equistimulation,” and then moved randomly in the vicinity of that line.

In order to move, DCs generate a traction force at their leading edge [10] by extending filopodia and binding them to a substrate by a set of “molecular clutches” [11]. So-called “clutches” are a general descriptor for a set of intracellular adapter proteins and a membrane-spanning integrin, which serve to connect the cytoskeleton to the extracellular space. Such a connection allows for force transduction from intracellular motors to the extracellular matrix as integrin-ligand bonds are stretched. This causes a force imbalance between the two ends of the cell and ultimately cellular motion. Previously, Chan and Odde modeled filopodial traction force generation in neuronal filopodia using stochastic binding and unbinding of molecular clutches [12]. Bound clutches were simplified to Hookean springs that would be stretched by the retrograde flow of the F-actin bundle to which they were attached. This model correctly predicted the effects of varying mechanical stiffnesses of substrate on filopodial movement in forebrain neurons. These findings were later expanded on by Bangasser *et al*. to simulate random migration of neurons [13]. They were able to predict the existence of an “optimum stiffness” for force transduction for different types of cells. However, the focus of these models was on force generation and neither allowed for modifications of a cell’s external chemical environment. Although originally proposed for neurons, the model presented by Chan and Odde is general enough to be applied to the migration of many cell types, including DCs. Our lab has previously measured the forces of DC filopodia pulling on micropost array detectors to be 0.5 nN [10], which will allow us to calibrate the model of filopodial dynamics to DC motility.

Here, we present a model of DC migration, which captures the role of receptor occupancy and signaling in directional motion. We integrate a model of filopodial force dynamics with a simple model of cell signaling that depends on receptor occupancy and a realistic phenomenological model for the placement of filopodia based on signal magnitude and direction. Filopodia are placed stochastically around the cell periphery, and the formation and breakage of adhesion is done using the Adhesive Dynamics methodology developed in our laboratory [14]. By combining these elements, we can recreate DC random and directed motility, and can predict the behavior of DCs in gradients and counter gradients of chemokine.

## Methods

**Fig 2:**
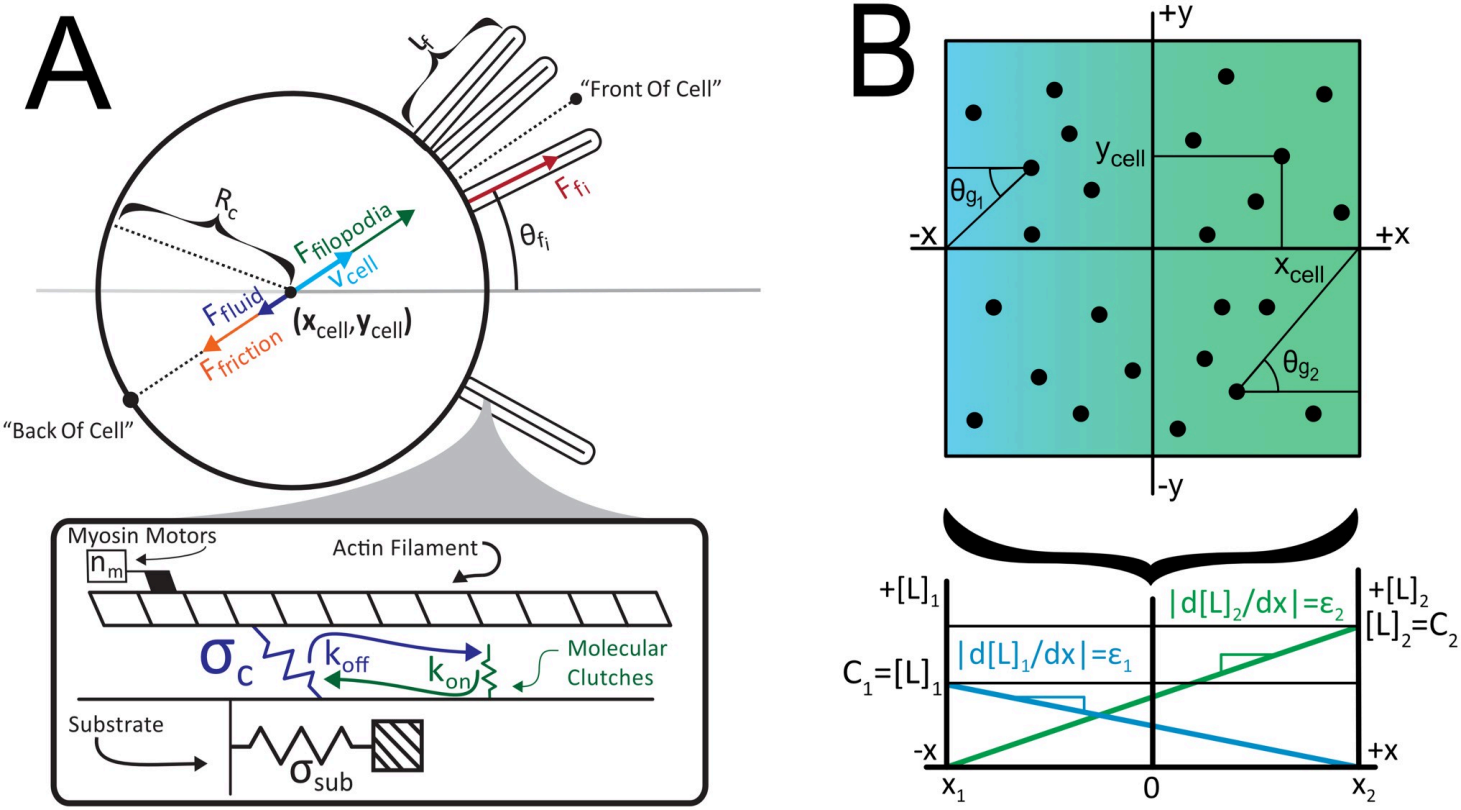
Schematic of the model. A) Filopodia are modeled as a set of molecular clutches that reversibly bind to an F-actin filament undergoing retrograde flow. These clutches allow force transduction and deformation of the substrate. Each cell is modeled as a circle of radius R_c_. The forces of all filopodia, F_*f*i_, are summed to a net force F_filopodia_ (green), which is balanced by resistive forces F_friction_ (orange) and F_fluid_ (purple) to produce a velocity v_cell_ (light blue). Also note position of the “front” and “back” of cell. B) Cells are simulated in linear, time-invariant gradients, each with a slope -ε_i_ and a maximum concentration C_i_. Each gradient is oriented at an angle θ_gi_ for the i^th^ gradient.

The basic schematic of our model is shown in Fig 2. DCs extend filopodia, which engage the substrate and generate a traction force, which by Newton’s third law also act on the body force on the cell. By force balance, the DC will move if it sees a differential force. The placement of the filopodia is determined by sampling a signaling function, which depends on receptor occupancy and the intracellular amplification of signals.

### Modeling clutch dynamics

In modeling clutches and filopodia, we have adopted the stochastic model of Chan and Odde [12], with some minor modifications. First, we calculated the probability of a clutch disengaging based on Adhesive Dynamics [14,15], and second, when a clutch disengages, the force it had exerted on the substrate is distributed to the rest of the engaged clutches. Otherwise, the method is identical to that of Chan and Odde and is only briefly summarized here.

Molecular clutches are modeled as Hookean springs with a spring constant (σ_clutch_) which can reversibly engage a filopod’s F-actin bundles with rate constants k_on_ and k_off_ (see Fig 2A). Because a time step on the order of the integrin bonding time is used, clutches are allowed to either engage or disengage the F-actin filament only once. As a result, the probability that a particular clutch will change its state in a given time step is given by:

$$P=1-e^{-k\Delta t}$$ (1)

where k is a pseudo rate constant for the reaction, in this case either k_on_ or k_off_ for binding or unbinding, respectively [14]. In the case of k_on_, integrins are assumed to be in excess. Once attached, clutches are stretched by the retrograde flow of the F-actin bundle and begin building up a force according to Hooke’s law. Imposing forces on the clutch in this manner weakens the bond that joins them and leads to an exponential increase in k_off_ as given by Bell’s Law [16]:

$$k_{off}^{*}=k_{off}(e^{\frac{F_{c}}{F_{b}}})$$ (2)

where F_b_ is the characteristic breaking force of the integrin-ligand bond and F_c_ is the force exerted by a single clutch.

### Filopodia and substrate

Filopodia are modeled as a set of myosin II motors pulling on a single F-actin bundle (Fig 2A). Each filopod pulls on a substrate with a spring constant σ_sub_, which represents the elastic nature of the surfaces they move on. Every time step, the substrate’s displacement from equilibrium is given by a simple force balance between forces exerted by the clutches and forces exerted by the substrate. This strain causes the substrate to exert a force counter to the motors, slowing them from their maximum velocity. In this case, the rate of retrograde flow is assumed to slow down by a linear force-velocity relationship given by

$$v_{r}=v_{u}(1-\frac{\sigma_{sub}\Delta x_{sub}}{n_{m}F_{s}})$$ (3)

where v_u_ is the unloaded motor/retrograde flow velocity, n_m_ is the number of motors, and F_s_ is the stalling force of a single motor. Once this retrograde velocity is calculated, all engaged clutches are stretched by v_r_Δt, and all clutches are permitted to both stretch and possibly unbind, as dictated by Eq 1. If a clutch had a nonzero stretch, then disengaged, the force it had exerted cannot simply disappear. Thus, after all clutches have been moved by the retrograde velocity of the F-actin bundle, the forces of all previously-stretched clutches are equally distributed to the n_eng_ remaining engaged clutches. This induces a further stretch Δx_d_ in all engaged clutches, which is given by

$$\Delta (\Delta x_{c})=\Delta x_{d}=\frac{\sum_{1}^{n_{d}}\Delta x_{c_{i}}}{n_{eng}}$$ (4)

where n_d_ is the number of clutches that have disengaged in a given time step and Δx_ci_ is the displacement of the i^th^ clutch from equilibrium. A demonstration that our adaptation gives the same dynamics described by Chan and Odde [12] is given in section 2 of the supplement of this paper (S1 Text).

### Cellular motion

While others have articulated the mathematical principles of filopodial clutch dynamics and force generation [12,14,15], we expanded on this work by integrating these filopodia into a cell. The idea of combining multiple filopodia on a single cell is similar in principle to that described by Bangasser *et al*., although we are now applying it to DC motion. The main part of each cell body is modeled as a circle possessing a number of filopodia, with the i^th^ filopod oriented at an angle θ_fi_ from the y = 0 axis, as shown in Fig 2A. We assume these are normally distributed around a central angle θ_mean_, which is further determined by the strength of signaling (see below).

Every time step, after all filopodia have calculated substrate displacement values, the cell calculates the net force exerted by the i^th^ filopod as F_fi_ = σ_sub_Δx_subi_. These forces are summed to obtain a net filopodial force. Countering this filopodial force are two resistive forces: fluid resistance and “molecular friction” caused by the continuous formation and breakage of clutch bonds between the cell body and the substrate. These two forces are both velocity-dependent. Because of the small Reynolds number of the system, all acceleration is assumed to be negligible, and the balance of forces is given by:

$$F_{filopodia}+F_{fluid}+F_{friction}=0$$ (5)

In calculating fluid resistance, the cell is approximated as a uniform sphere of radius R_c_ moving through a fluid of viscosity μ at a velocity v_c_ and thus experiences a resistive force given by Stokes Law:

$$F_{fluid}=-6\pi {\mu R}_{c}v_{c}$$ (6)

To estimate the molecular friction, the cell is modeled as a circle of radius R_c_ moving at a velocity v_c_ over a two-dimensional substrate. In a time interval Δt, such a cell would pass over an area ΔA = (2R_c_)(vΔt). The circle is taken to have a constant receptor density δ = dn/dA. Thus, in passing over such an area, the cell must break Δn = δ2R_c_vΔt receptor-ligand bonds. Increasing the number of receptor-ligand bonds broken in a given time step likewise increases the force against which the cell must work by γ = dF/dn. This means γ is reasonably approximated by the mean breaking force of an integrin-ligand bond. Thus, the resistive force can be approximated as

$$F_{friction}\approx -2\frac{dF}{dn}\frac{dn}{dA}\;R_{c}v_{c}\Delta t=-(2\gamma \delta R_{c}\Delta t)v_{c}=-k_{fr}v_{c}$$ (7)

Using these approximations for F_fluid_ and F_friction_, Eq 5 can be solved for velocity as

$$v_{c}\in C=\frac{F_{filopodia}}{6\pi \mu R_{c}+k_{fr}}$$ (8)

Once a cell’s velocity has been calculated, its x and y position are adjusted by v_c_Δt. Note that in our simulations, k_fr_>>6πμR_c_, but the fluid resistive force has been retained to allow the model to be generalized to situations where this is not the case. Changes in direction come from changes in the filopodial dynamics and orientation (see below).

### Calculation of frictional force

In order to provide accurate results, it was necessary to estimate the frictional force experienced by cells. Others have done this by tracking individual clutches on the cell body [13], but this can be simplified for the time scales of cellular motion by Eq 7. Friction was calculated assuming a substrate coated with fibronectin, as we would compare model predictions to experiments done on murine DCs migrating on glass slides coated with fibronectin [17].

We directly measured the radius of the murine dendritic cells used in this experiment from video images (see Fig 1) as R_c_ = 5μm. We assumed all bonds would break at the mean breaking force of a fibronectin-integrin bond of 85 pN [18,19]. Moreover, we assumed that the cell had a constant receptor density of 490 μm^-2^ [20,21]. Finally, we used a time step of 5 ms, which allows us to ignore viscous effects of the substrate, and which is small relative to the time scales of cellular motion. Using these, we calculated the frictional coefficient to be 2.1*10^6^ μg·s^-1^ (see S1–S4 Tables for a table of all constants used). This value was used throughout all simulations.

### Linear gradients

All simulated chemotaxis data have been collected assuming linear, temporally invariant chemokine gradients. There are several reasons for this, primarily that it allows for analysis of DC decision-making in the absence of potentially confounding variables, such as time and position dependence of gradient slopes (as might be seen in point-source diffusion, for example). This also allowed us to compare results with past experiments that have used linear gradients [8].

Linear gradients are recreated in simulation by placing a line of high concentration at an x position x_gradient_ far beyond the region the cell could conceivably reach in the time it is given. These gradients decrease linearly with a slope -ε from a maximum ligand concentration of C_0_. Using these parameters, the concentration for a cell at x position x_cell_ is given by

$$C(x_{cell})=C_{0}-\varepsilon |x_{cell}-x_{gradient}|$$ (9)

Eq 9 could be readily modified to allow for time-dependence, and nonlinearity in gradients, which would allow the model to work with almost any chemical environment created experimentally.

### Cellular signaling

Cells will reorient filopodia based on their chemical environments. We modeled this behavior by first calculating “individual signals” from each gradient, which are composites of three values: the concentration-based signal strength of the i^th^ gradient, denoted S_gi_, its direction of strength, denoted θ_gi_, and its “intrinsic” signal strength, denoted a_gi_. These are used to create an “overall signal” which can be used to reorient filopodia. The details of the signal transduction network inside the cell are undoubtedly much more complex than this, but this simple approximation will inform us about the relative signaling strength of the simulation.

The signal from each chemokine receptor, S_gi_, derives from the its fractional occupancy *f* ([L]). The fractional receptor occupancy can be thought of as either the fraction of receptors that are occupied in a given local area, or as the probability that a randomly selected receptor within an area will be bound to a ligand. We assumed that the cell’s receptors are in chemical equilibrium with their surroundings. This means that the fractional occupancy is given by

$$f(\left[L\right])=\frac{\left[L\right]}{[L]+K_{d}}$$ (10)

where [L] is the ligand concentration and K_d_ is the dissociation constant of the ligand [9]. S_gi_ is defined as the difference in fractional occupancy across the “front” and “back” of the cell. The “front” of the cell (FOC) is taken to be the position at the tip of a filopod with a length *l*_*f*_ oriented along the direction of the cell’s motion (Fig 2A). The “back” (BOC) is defined as the rearmost part of the cell along its direction of motion. Thus, S_gi_ may be mathematically defined as

$$S_{g_{i}}=|\frac{C(FOC)}{C(FOC)+K_{d}}-\frac{C(BOC)}{C(BOC)+K_{d}}|$$ (11)

where C(FOC) and C(BOC) can further be described by Eq 10 in terms of R_c_, *l*_*f*_, and the cell’s direction of motion φ:

$$C(FOC)=C_{0}-\varepsilon |\{x_{cell}+[R_{c}+l_{f}]\text{cos}\varphi \}-x_{gradient}|$$ (12)

$$C(BOC)=C_{0}-\varepsilon |\{x_{cell}-R_{c}\text{cos}\varphi \}-x_{gradient}|$$ (13)

θ_gi_ is defined as the angle made by a horizontal vector going from (x_cell_, y_cell_) to (x_gradient_, y_gradient_ = 0) (Fig 2B). This is distinct from the direction of the cells motion φ, which refers to the actual motion of the cell, rather than where the gradient is directing it to go. This distinction is important when modeling counter gradients. Finally, α_gi_ is used to account for the strong cellular “preference” DCs exhibit for some chemokines over others [8]. Until the physical basis for such preference is better understood, such constants must be set phenomenologically.

### Rearrangement of filopodia

Cells periodically rearrange individual filopodia. We assume this rearrangement is done on an average rearrangement time scale, τ. At the beginning of every simulation, τ is randomly assigned according to a narrow but normal distribution with a mean μ = T and standard deviation σ = T/10. T is a simulation-wide parameter meant to approximate how often murine DCs rearrange their filopodia. Once τ time steps have passed, a single filopod is reoriented towards a new angle, a new filopodial rearrangement time is calculated, and the cycle begins again.

Once a cell has determined it needs to reorient a filopod, it must calculate a new angle θ_new_ at which to place it. This calculation is based on a normal distribution of possible angles, and thus a cell must first calculate a mean angle and standard deviation of that angle, based on the magnitude of the signal the cell senses. This, in turn, requires the calculation of a composite signal strength S_c_ and a composite angle of stimulation θ_c_. They are both weighted averages, and are given for n_g_ gradients as

$$S_{c}=\frac{\sum_{1}^{n_{g}}S_{g_{i}}\alpha_{g_{i}}}{\sum_{1}^{n_{g}}\alpha_{g_{i}}}$$ (14)

$$\theta_{c}=\frac{\sum_{1}^{n_{g}}\theta_{g_{i}}S_{g_{i}}\alpha_{g_{i}}}{\sum_{1}^{n_{g}}S_{g_{i}}\alpha_{g_{i}}}$$ (15)

S_c_ is then scaled appropriately based on a maximum value of S_c_ within the positional bounds of the simulation, S_max_, and mapped to a standard deviation (of a normal distribution) σ_θ_ between a minimum and maximum value as such:

$$\sigma_{\theta }=-[\frac{\sigma_{max}-\sigma_{min}}{\pi }]{\text{tan}}^{-1}\left[\pi \text{tan}\left(\frac{\pi S_{c}}{S_{max}}-\frac{\pi }{2}\right)\right]+\frac{\sigma_{max}+\sigma_{min}}{2}$$ (16)

The above phenomenological function was crafted to give a graph with the following features: 1) mapping S_max_ to σ_min_, and 0 to σ_max_, thus giving weak signals a lack of directionality, 2) exhibiting an inflection point at S_c_ = S_max_/2, so that the function has reasonably positive curvature at S_c_ < S_max_/2 and negative otherwise, 3) having a useful domain of (0, S_max_) and a range of (σ_min_, σ_max_).

In the case of gradients varying only in the x direction, θ_c_ is converted to a new angle θ_mean_ by normally distributing it around a mean of either μ = π or μ = 0, depending on the quadrant in which θ_c_ lies. θ_mean_ is given by a normally distributed random variable N(μ,σ), where μ is the mean of the normal distribution, and σ is the standard deviation.

$$\theta_{\text{mean}}=\{\begin{array}{c} \text{N}\left(\mu ,\sigma \right)=\text{N}\left(0,\;\sigma_{\theta }\right)\;\text{if}\;\theta_{\text{c}}\;\text{is}\;\text{in}\;\left(-\frac{\pi }{2},\frac{\pi }{2}\right)\\ \text{N}\left(\mu ,\sigma \right)=\text{N}\left(\pi ,\;\sigma_{\theta }\right)\;\text{if}\;\theta_{\text{c}}\;\text{is}\;\text{in}\;\left(-\pi ,\;-\frac{\pi }{2}\right)\cup \left(\frac{\pi }{2},\pi \right) \end{array}$$ (17)

Finally, once both a mean and standard deviation have been calculated, a filopod can finally be assigned an angle θ_new_ as a normal distribution with a mean μ = θ_mean_ and standard deviation σ = σ_θ_. In reorienting a filopod, all molecular clutch bonds are assumed to break, and the filopod must build up new attachments.

In this model, we have fixed the number of filopodia to be 4 (a reasonable number based on images in Fig 1). Of course, this can be varied, depending on the strength of the signal, but as will be seen, giving all DCs 4 filopodia captures the basic physics of motion quite well. Also, we set the length of filopodia to be a fixed number, L. This can be varied in future versions of the method.

## Results

### Filopodial force

We began by checking that the mechanics of filopodial force generation matches the value determined by past experiments. To calculate the average force per filopod, we logged the net force exerted by every filopod at every time step. After simulating 400 cells, we arrived at a mean filopodial force of 183 ± 94 pN. This is consistent with both past simulated results of ~250 pN for stiff substrates [estimated from data provided by 11]. It is also consistent with experiments done by Ricart *et al*., which showed the average force to be 550 ± 400 pN [10]. Any discrepancy between the results is likely due to Ricart’s use of micropost arrays, which have been shown to increase speed of cells [17], and thus likely the force exerted by individual filopodia. Our model also exhibited the same load-and-fail dynamics for softer substrates that have been reported by others [12], which is the leading cause of the large standard deviation of the net filopodial force (see S1 Text, section 1).

### Random motility

**Fig 3:**
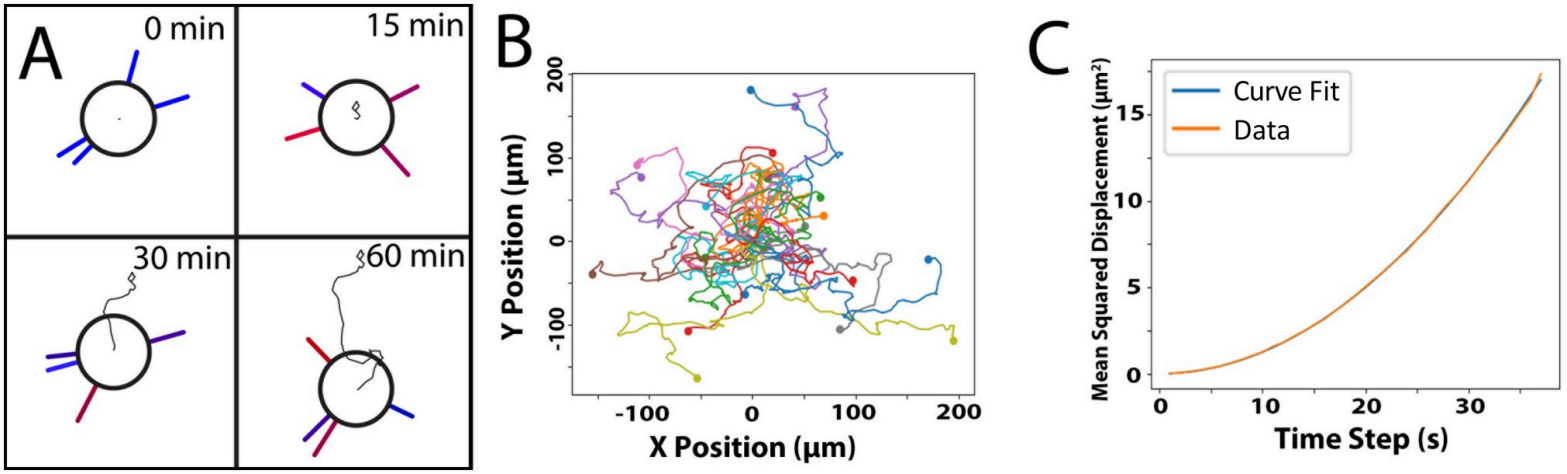
A) A typical path for a cell undergoing chemokinesis, where red filopodia exert large forces on the cell, and blue ones exert small forces. B) Scattergram of 25 cells undergoing chemokinesis. Dots represent the cell’s final position. C) Mean squared displacement vs. time. Trajectories accurately fit with the Dunn equation, from which a speed and persistence time can be elucidated.

We then simulated the motion of DCs in uniform fields of chemoattaractant and created scattergrams of the motion (Fig 3B). We could then extract the speed S and persistence time P from the time lag of the mean squared displacement. These values could be directly compared with available literature data. This represents a further check on our simulations.

Analysis of positional data gave speed values of 5.07 ± 0.07 μm·min^-1^ and persistence time values of 3.17 ± 0.46 min, which is in good agreement with the experimental values observed by our laboratory for the motion of DCs, which are of 4.00 ± 0.18 μm·min^-1^ and 4.88 ± 0.46 min, respectively [17]. The trajectories of individual cells are also in good qualitative agreement with those of actual DCs (Fig 3). Based on these results, our model appears to be able to model random motion well, exhibiting the proper behavior both at the filopodial and cellular level.

### Counter gradients and points of equistimulation

**Fig 4:**
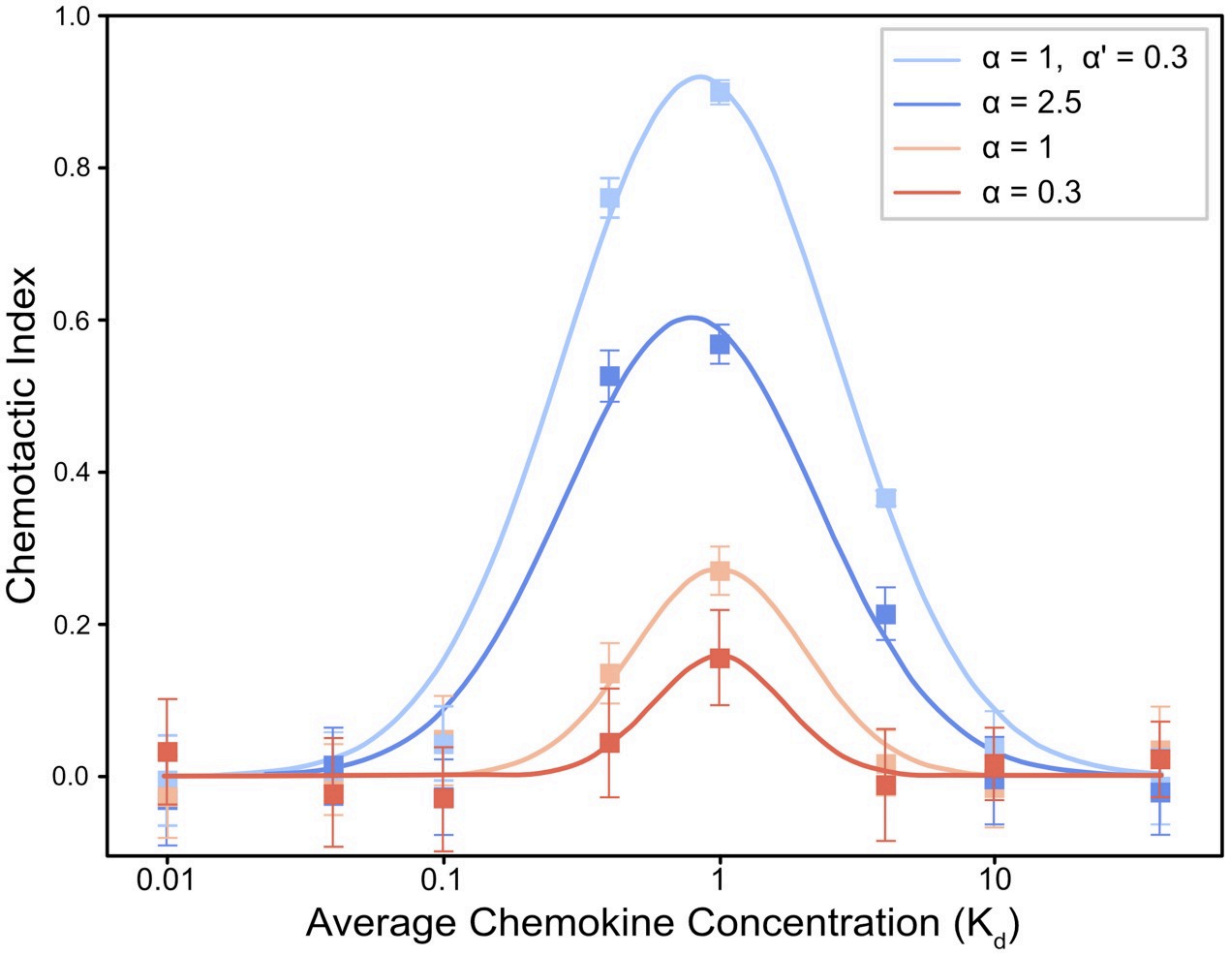
Plot of chemotactic index (CI) as a function of average chemokine concentration, shown for both three individual gradients and for two gradients working together. In all cases, the CI is a maximum when the average chemokine concentration is equal to the K_d_, while returning to zero at concentrations well above and below the K_d_. In addition, CI scales with relative strength of the gradient: stronger gradients lead to larger vales of CI, and also a wider range of concentrations over which CI is high. In the case of a coincident gradient, the effect of combining two gradients of relative strength of 1 and 0.3 is much greater than would be predicted by simply adding their CIs. Values are plotted as mean ± ME.

When exposed to a single gradient, simulated cells consistently migrated towards higher concentrations of chemokine. While doing so, they exhibited faster speeds and longer persistence times than cells undergoing random motion, indicating a chemotactic response. The degree of cellular response varied widely with both the relative strength of the gradient and its mean chemokine concentration, as shown in Fig 4. Gradients with higher relative strengths gave rise to a stronger response and higher chemotactic indices. Interestingly, however, we found that combining gradients gave rise to a higher chemotactic index than a simple sum of each chemotactic index would suggest. At constant relative strength, cellular response depended heavily on the mean chemokine concentration. Cells exhibited stronger responses when the mean concentration of chemokine was close to the K_d_ (Fig 4). If the mean concentration was much lower than the K_d_, the cell produced weak responses as a result of a general lack of chemokine (small fractional occupancy), while higher mean concentrations also produced weaker responses due to receptor saturation. As cells migrated farther up each gradient, resulting in increased average levels of chemokine, the strength of their chemotactic responses decreased. As a result, cells changed the direction of migration more frequently, causing a reduction in directional motion. Presumably, this is the result of receptor saturation disabling the cells’ ability to sense the gradient.

**Fig 5:**
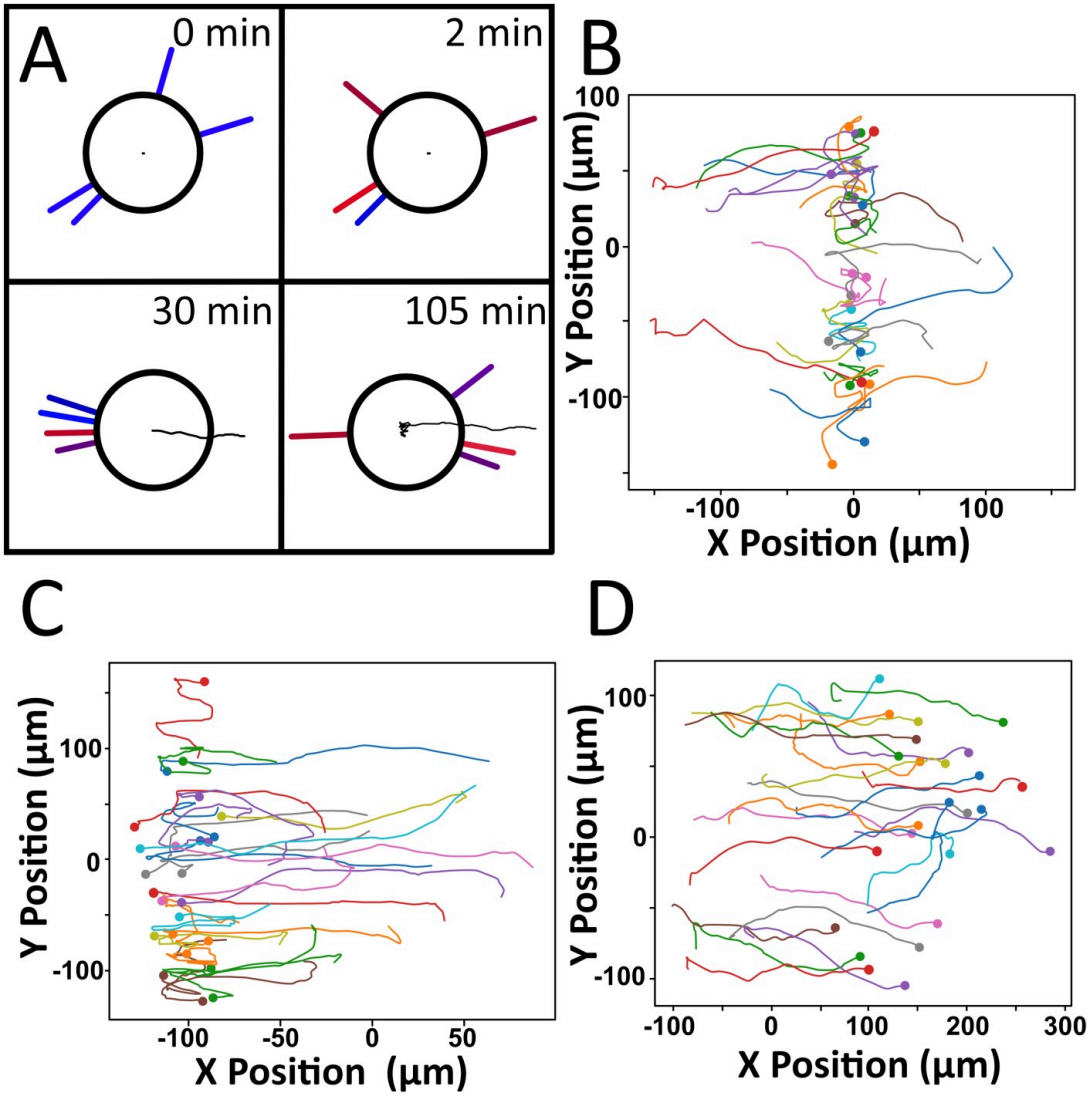
A) A typical path for a cell undergoing chemotaxis, illustrating 1) the typical time frame for a filopod to be reoriented, 2) the focused arrangement of filopodia while the cell is undergoing chemotaxis, 3) the formation of a single preferred x-value around which the cell doesn’t move, and 4) the disappearance of focus in filopodia once that line is reached. B) Representative positions of 25 cells clustering in a line, where both gradients have identical slopes, strengths, and K_d_ values. C) Cells do not have to cluster around x = 0, but can cluster at many other locations to reach their destination. D) If the counter gradient is too shallow, cells will not find an equilibrium position.

When exposed to counter gradients, cells generally exhibited one of three behaviors. In most cases, groups of cells found a single, well-defined line of equistimulation, with the group exhibiting a stable mean x position with typical standard deviations between 10μm and 30μm. This is similar to past experiments performed by our lab, in which cells along a line of equistimulation exhibited standard deviations of approximately 25μm [8]. Such lines of equistimulation are visually extremely distinct, as shown in Fig 5B and 5C. These lines were less well-defined in shallower gradients, as the region in which cell motion was random was wider. Cells in shallow gradients also exhibited relatively larger standard deviations of position. Even in this situation, cells exhibited the characteristic lack of net directional migration associated with a line of equistimulation. If one of the gradients became particularly weak relative to another, cells sometimes failed to exhibit any line of equistimulation, as shown in Fig 5D. This is equivalent to a limiting case in which one gradient becomes so flat as to provide no net directional signal, and migration resembled that of a single gradient.

**Fig 6:**
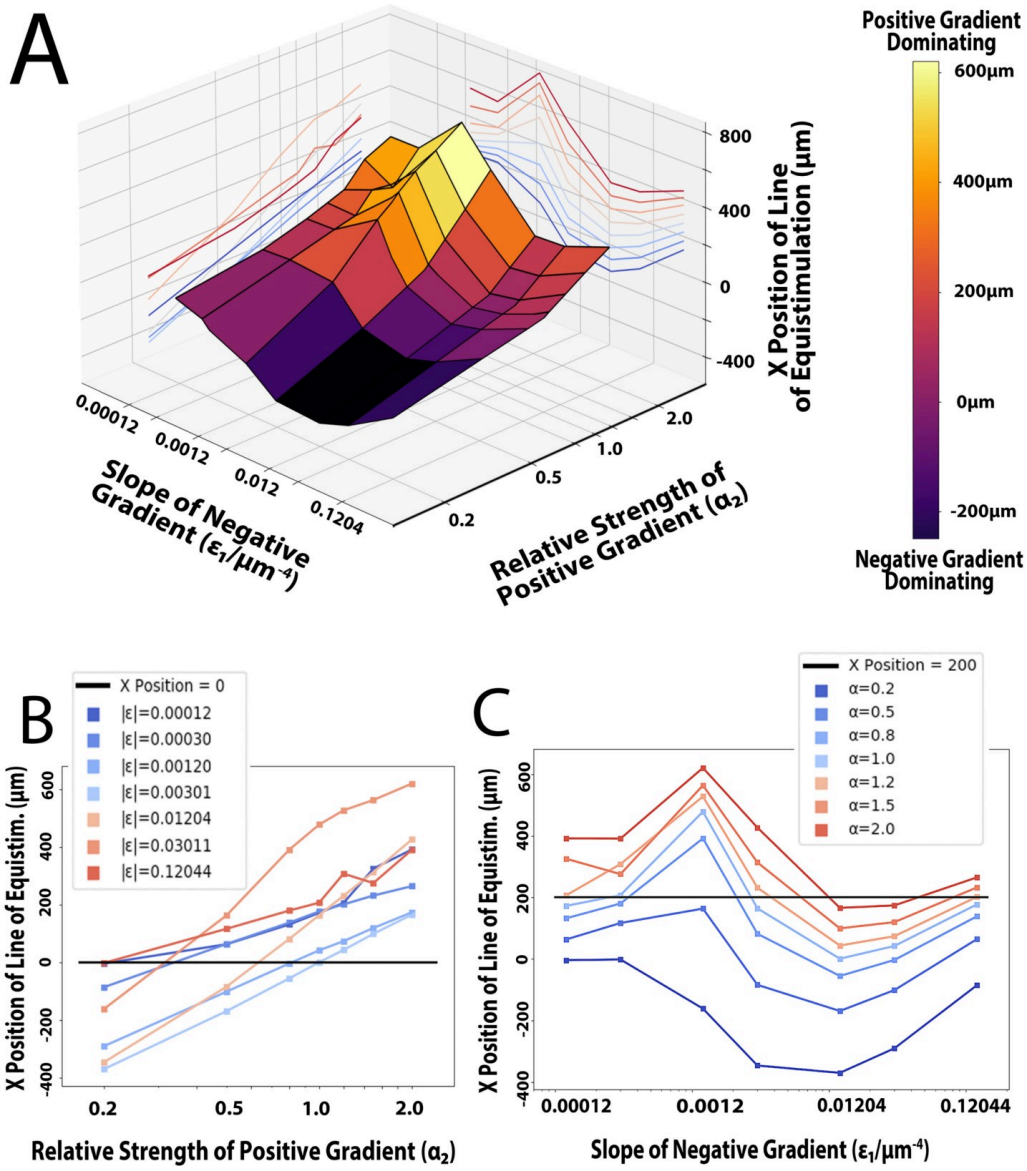
A) 3D plot of the conditions varied and their effects on the x position of the line of equistimulation. Note the peaks and troughs, which represent where the positive and negative gradient is most effective, respectively. Also note the relative flatness of the graph for sufficiently small slope values. B) Logarithmic plot of the relative strength of the positive gradient’s effects on the point of equistimulation, with the positive gradient’s slope (ε_2_) and average concentration held constant at 0.012044μm^-4^ and the K_d_, respectively. Each line represents a different slope (ε_1_) for the negative gradient. C) Logarithmic plot of the effects of varying the slope and average concentration of the negative gradient on the point of equistimulation, with the negative gradient’s relative strength (α_1_) held constant at 1. Each line represents a different relative strength of the positive gradient. Note 1) the constant-slope region when ε_1_ is negligible, 2) the maximum at ε_1_ = 0.0012044μm^-4^, corresponding to an average concentration equal to K_d_/10, indicating the negative gradient is least effective when this is the case 3) the minimum at ε_1_ = 0.012044μm^-4^, corresponding to an average concentration equal to the K_d_, indicating the negative gradient is most effective when this is the case.

Here, we have mostly studied outcomes where cells exhibit a stable line of equistimulation. We did so by simulating similar conditions to those examined by Ricart [8], with one gradient increasing as the x co-ordinate decreased–the “negative” gradient–and another increasing as the x co-ordinate increased–the “positive” gradient. We set the relative strength of the negative gradient to 1, and the slope of the positive gradient such that concentration values ranged from 0-2K_d_. We chose to keep these factors constant so as to avoid a gradient having both a shallow slope and a low relative strength, thus effectively removing its effect on cells. Using these two constant parameters, we varied the slope of the negative gradient (ε_1_) and the relative strength of the positive gradient (α_2_), and recorded the mean x position at which cells eventually exhibited no net x-directional motion. Here, we refer to this mean x position of cells in equilibrium as the x position of the line of equistimulation. The results of this computer experiment are plotted in Fig 6. Combinations of low ε_1_ and high α_2_ values produced significant motion towards one pole, as shown by the sharp peak in Fig 6A. Conversely, low α_2_ and high ε_1_ values produced nearly the opposite effect, strongly stimulating cells to go towards the negative pole, as shown by the deep valley in Fig 6A. When only varying α_2_, we found that increasing the relative strength of a gradient was found to increase its effectiveness at directing cells, but did so logarithmically (Fig 6B). We curve-fitted the data (average R^2^ of 0.96), and used our lab’s previous findings [8] to assign numerical values to the strengths of CCL19, CCL21, and CXCL12. Normalizing all strengths around α_CCL19_ = 1, we gave CCL21 and CXCL12 strengths of 0.28 and 0.30, respectively. This assignment of a relative strength to a gradient can also be seen graphically by computing the x-intercepts of the constant-slope (constant average concentration) lines of Fig 6B. At the x-intercept, the negative gradient (with the specified slope) and the positive gradient (with the calculated relative strength) attract a cell with equal strength.

We found that the mean x position stayed roughly constant for sufficiently low values of a gradient’s slope, presumably because cells have difficulty distinguishing it from a constant chemokine field. Once ε_1_ was increased enough that cells could sense the gradient, it exhibited a minimum of effectiveness when the average concentration was roughly K_d_/10, and a maximum of effectiveness when the average concentration was equal to the K_d_, as shown by the peaks and valleys of Fig 6C, respectively.

At every constant α_2_, there were often multiple slopes that would cause cells to localize at a given x position (for greater detail on producing a line at x = 0, see S2 Fig). Consider the case where cells line up at x = 200 (the black line in Fig 6C), and α_2_ = 1.2. When the slope is almost zero, cells will line up at x = 200 because they can barely sense the negative gradient, and are almost solely compelled by the positive gradient until the signal from the negative gradient increases. When ε_1_ is increased such that the average concentration of the negative gradient moves beyond the minimum-effectiveness K_d_/10, it is possible to compel cells to line up at x = 200 again. This is because cells are finally able to sense the negative gradient better than they could before, getting closer to the balance between there being enough ligand to perceive the gradient, but not too much as to saturate receptors. When the slope is increased such that the average concentration is equal to the K_d_, the negative gradient is at its most effective. Further increases to ε results in less directional motion, as the average chemokine concentration prevents the cell from sensing the gradient.

## Discussion

Here, we have presented a model for dendritic cell directional motion and chemotaxis. We have been able to replicate several experiments, ranging from filopodial force dynamics to cellular motion and chemotaxis. We can successfully simulate the force of an individual filopod and find our results are consistent with both past experiments and past simulations. Moreover, we have been able to numerically quantify cellular preference for certain chemokines. Our model can predict where cells will likely cluster based on both their chemical composition and steepness of gradient. More importantly, the model presented here can be applied to an arbitrary set of experimental conditions and chemokine gradients. This could potentially open the door to modeling the same chemical environment to which DCs are exposed in the body. Extension of the model to three-dimensional environments would only require some topological information about the environment, and the models of motion could be adapted. This potentially would allow the simulation of DC migration from the periphery to secondary lymphoid organs, which in turn could be used to better understand antigen trafficking as a whole. This understanding could allow for the creation of more computationally efficient models of antigen trafficking once the population dynamics of DC migration are better understood. Further extensions of the model would allow for incorporating different material properties of the material, such as migration through regions of different compliances, or regions of gradients of stiffness (so called durotaxis).

While the model faithfully recreates the notion that DCs can find lines of equistimulation, there are two phenomenological features of the model that can be improved with additional biological information. First is the manner in which the signal of receptor occupancy is amplified. Here we choose to model the behavior by a simple amplification function that is the product of receptor occupancy that seems to capture the essential essence of the signaling response. An increased understanding of the interactions between the intracellular signaling cascades downstream of receptor-ligand binding might allow for estimation of the amplification coefficients *a priori*. Future models could incorporate this signaling behavior, provided parameters could be estimated to get an accurate measure of signaling strength. We have also chosen to approximate how cells decide to place their filopodia. As illustrated in Fig 1, in a strong chemotactic field, DCs orient their filopodia to one pole; in random migration, the filopodia are organized randomly. The cells must integrate the signaling information it receives to decide where the filopodia are placed, and this must be done by spatially organizing actin polymerization centers around the cell periphery in response to the signal. We lump the details of this organization into a simple functional form that indicates the most likely angle of orientation in response to the gradient. Although certain aspects were recreated phenomenologically, this is quite commonplace in cell motility modeling and captures the zeroth order essence of the response (see for example, Iglesias et al. [22]). Future work can include mechanisms of local actin organization and polymerization to allow for spontaneous filopod formation.

## Supporting information

S1 Fig Graph of the variation of the substrate displacement (y axis, in nanometers) from equilibrium as time (x axis, in seconds) progresses. The blue curve represents a relatively compliant substrate, while the red curve represents a relatively stiff substrate.
(TIFF)

S2 Fig Combinations of relative strength and slope that lead to no net directional motion (i.e. a line of equistimulation at x = 0). Using the data and parameters from Fig 6, we have approximated a smooth curve that shows all combinations of ε_1_ and α_2_ that should lead to approximately no directional motion, and thus which lead to equally effective gradients. Above the line, cells will move in the negative direction; below the line, cells will move in the positive x-direction.
(TIFF)

S1 Table Filopodial parameters. Definitions, values and sources of filopodial parameters.
(DOCX)

S2 Table Cellular parameters. Definitions, values, and sources of all cellular parameters.
(DOCX)

S3 Table Gradient parameters. Definitions, values, and sources of all gradient parameters.
(DOCX)

S4 Table Simulation-wide parameters. Definitions, values, and sources of all system wide parameters.
(DOCX)

S1 Video Animation of chemokinesis. (MP4)

S2 Video Animation of chemotaxis. (MP4)

S1 Code Commented DC simulation code. (TXT)

S1 Text A single file that contains a description of methods, the supplemental figures, tables and references. (DOCX)
