# Supplementary figures and images for: A simulation of the random and directed motion of dendritic cells in chemokine fields
Source: PLoS Comput Biol. 2019 Oct 7;15(10):e1007295. doi: 10.1371/journal.pcbi.1007295 (PMC6797211; doi:10.1371/journal.pcbi.1007295)

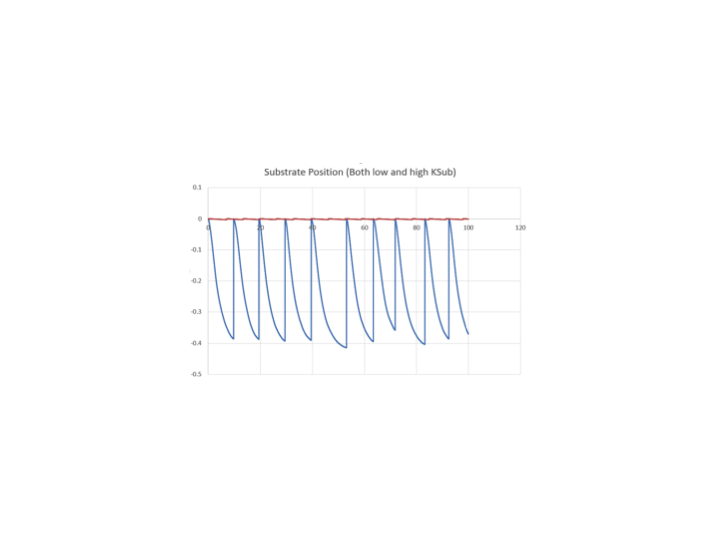

Supplement: S1 Fig — The blue curve represents a relatively compliant substrate, while the red curve represents a relatively stiff substrate. (TIFF) [file pcbi.1007295.s001.tiff]

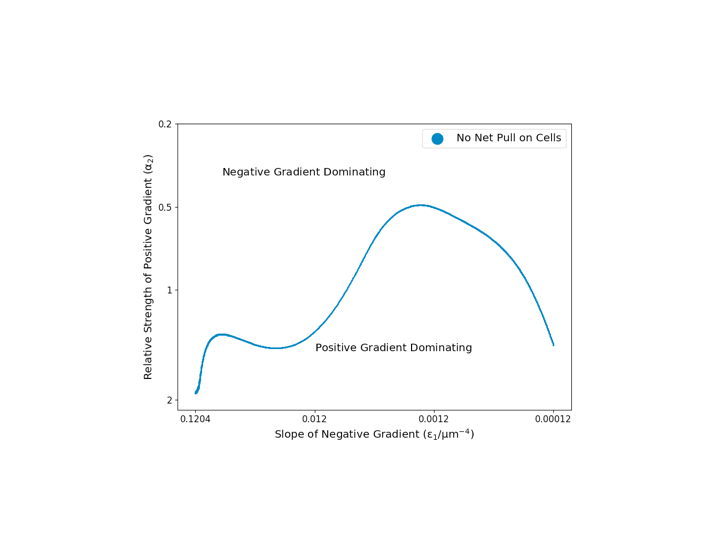

Supplement: S2 Fig — Using the data and parameters from Fig 6, we have approximated a smooth curve that shows all combinations of ε1 and α2 that should lead to approximately no directional motion, and thus which lead to equally effective gradients. Above the line, cells will move in the negative direction; below the line, cells will move in the positive x-direction. (TIFF) [file pcbi.1007295.s002.tiff]
