# Supplementary material for: A simulation of the random and directed motion of dendritic cells in chemokine fields
Source: PLoS Comput Biol. 2019 Oct 7;15(10):e1007295. doi: 10.1371/journal.pcbi.1007295 (PMC6797211; doi:10.1371/journal.pcbi.1007295)
Supplement: S1 Table — Definitions, values and sources of filopodial parameters. (DOCX) [file pcbi.1007295.s003.docx]

Table S1. Filopodial Parameters. Definitions, values and sources of filopodial parameters.

| Parameter Symbol | Description | Value | Source |
| --- | --- | --- | --- |
| $k_{\mathrm{on}}$ | Pseudo rate constant (1^st^ order) for molecular clutch engaging F-actin bundle | 1 s^-1^ | Chan, Odde, et al., 2008, 1 s^-1^ [2] |
| $k_{\mathrm{off}}$ | Pseudo rate constant (1^st^ order) for engaged molecular clutch to disengage | 0.02 s^-1^ | Lele et al., 2008, 0.01-0.1 s^-1^ [3] |
| $F_{b}$ | Characteristic breaking force of a clutch-F-actin bond | -2 pN | Jiang et al., 2003, 2pN [4] |
| $v_{u}$ | Unloaded retrograde velocity of F-actin bundle | -120 nm/s | Chan, Odde, et al., 2008, 120 nm/s [2] |
| $\sigma_{\mathrm{sub}}$ | Spring constant of deformable substrate | 80 nN/μm | Chan, Odde, et al., 2008, order of nN/μm [2] |
| $n_{m}$ | Number of myosin motors powering the retrograde flow of a single F-actin bundle | 120 | Adjusted upward from Chan, Odde, et al. [2] to match filopodial force |
| $F_{s}$ | Stalling force of an individual myosin motor | -2 pN | Molloy et al., 1995, [5] |
| $n_{c}$ | Approximate number of molecular clutches associated with a filopod | 40 | Adjusted downward from Chan, Odde, et al. to match retrograde flow rates [2] |
