# Supplementary material for: A simulation of the random and directed motion of dendritic cells in chemokine fields
Source: PLoS Comput Biol. 2019 Oct 7;15(10):e1007295. doi: 10.1371/journal.pcbi.1007295 (PMC6797211; doi:10.1371/journal.pcbi.1007295)
Supplement: S2 Table — Definitions, values, and sources of all cellular parameters. (DOCX) [file pcbi.1007295.s004.docx]

Table S2. Cellular Parameters.

| Parameter Symbol | Description | Value | Source |
| --- | --- | --- | --- |
| $R_{c}$ | Typical dendritic cell radius | 5 μm | Directly measured from videos of DCs [6] |
| $\mu$ | Viscosity of water (taken to be similar to most aqueous solutions) | 0.001 Pa s | Well-known physical constant |
| $\gamma$ | Characteristic breaking force of fibronectin-integrin bond to approximate molecular friction | 85 pN | Li et al., 2003, 85pN [1] |
| $\delta$ | Surface integrin density of DCs | 490 μm^-2^ | Patla et al., 2010, 490 μm^-2^ [7] |
| $k_{\mathrm{fr}}$ | Velocity dependent force constant (F=kv) due to molecular friction | 2.1*10^6^ μg s^-1^ | Estimated based on above parameters (equation 7) |
| $\sigma_{\max}$ | Maximum standard deviation for Normal distribution according to which filopodia will be rearranged | π rad | Chosen so all possible angles were within 1 SD of the mean |
| $\sigma_{\min}$ | Minimum standard deviation for Normal distribution according to which filopodia will be rearranged | 0.4 rad | Directly measured based on videos of DCs undergoing chemotaxis [6] |
| $S_{\max}$ | Approximate maximum, dimensionless “signal” quantity a cell receives. | 0.005 | Calculated based on a cell in a chemokine gradient at a concentration equal to the K_d_ |
| $n_{f}$ | Number of filopodia associated with each cell | 4 | Measured based on videos of DCs undergoing chemotaxis/chemokinesis |
