# Supplementary material for: A simulation of the random and directed motion of dendritic cells in chemokine fields
Source: PLoS Comput Biol. 2019 Oct 7;15(10):e1007295. doi: 10.1371/journal.pcbi.1007295 (PMC6797211; doi:10.1371/journal.pcbi.1007295)
Supplement: S3 Table — Definitions, values, and sources of all gradient parameters. (DOCX) [file pcbi.1007295.s005.docx]

Table S3. Gradient Parameters.

| Parameter Symbol | Description | Value |
| --- | --- | --- |
| $\varepsilon_{1}$ | Slope of negative gradient, which increases with decreasing x values | 3: 0  4: {0.2pM/μm, 0.5pM/μm, 2pM/μm, 5pM/μm, 20pM/μm, 50pM/μm, and 200pM/μm}, with C_avg_={K_d_/100, K_d_/40, K_d_/10, K_d_/4, K_d_, 2.5K_d_, and 10K_d_}, respectively.  5B: 0.02nM/μm; C_avg_=K_d_  5C: 0.05nM/μm; C_avg_=2.5K_d_  5D: 0.002nM/μm; C_avg_=0.1K_d_  6: {0.2pM/μm, 0.5pM/μm, 2pM/μm, 5pM/μm, 20pM/μm, 50pM/μm, and 200pM/μm}, with C_avg_={K_d_/100, K_d_/40, K_d_/10, K_d_/4, K_d_, 2.5K_d_, and 10K_d_}, respectively. |
| $K_{d_{1}}$ | K_d_ of negative gradient | 6.022$molecules\cdot\mu m^{-3}=10nM$ [6] |
| $\alpha_{1}$ | Intrinsic strength of negative gradient, representing strength of cellular preference for the negative gradient. | Chemotactic simulations: $\alpha_{1}=1$  Chemokinetic simulations: $\alpha_{1}=0$ |
| $\varepsilon_{2}$ | Slope of the positive gradient, which increases with increasing x values | Chemotactic simulations: $\varepsilon_{2}=0.02nM;C_{\mathrm{avg}}=K_{d}$  Chemokinetic simulations: $\varepsilon_{2}=0$ |
| $K_{d_{2}}$ | K_d_ of positive gradient | $6.022 molecules\cdot\mu m^{-3}=10nM$ [6] |
| $\alpha_{2}$ | Intrinsic strength of positive gradient | 3: 0  4: positive gradients had strengths of 0.3, 1, and 2.5, as well as a combination of 1 and 0.3  5B: 1  5C: 0.5  5D: 1.5  6: 0.2, 0.5, 0.8, 1, 1.2, 1.5, 2 |
