# Supplementary material for: A simulation of the random and directed motion of dendritic cells in chemokine fields
Source: PLoS Comput Biol. 2019 Oct 7;15(10):e1007295. doi: 10.1371/journal.pcbi.1007295 (PMC6797211; doi:10.1371/journal.pcbi.1007295)
Supplement: S4 Table — Definitions, values, and sources of all system wide parameters. (DOCX) [file pcbi.1007295.s006.docx]

Table S4. Simulation-Wide Parameters.

| Parameter Symbol | Description | Value | Source/Justification |
| --- | --- | --- | --- |
| $\Delta t$ | Time step used for simulation | 5 ms | Consistent with past simulations at the filopodial level [2], and much smaller than the timescales of cellular motion. |
| $\mathrm{TicksPerSample}$ | Number of simulation ticks allowed to pass before recording positions, velocities, etc. | 200 | Chosen a) so that data would be taken once every second of simulated time, and b) to reduce memory usage approximately 200-fold, so as to allow for longer simulations. |
| $\mathrm{SimulationLength}$ | Number of ticks in a simulation | 3*10^6^ or 7.2*10^5^ | Chosen to allow a long enough time for cells to reach equilibrium positions; 3,000,000 ticks represents approximately 4.1 hours, 720,000 a time of 1 hour exactly. |
| $\mathrm{NumberOfCells}$ | Number of cells simulated | 1 or 25 or 50 | Varied based on what was trying to be simulated; most simulations used 25 cells, but Figures 3A and 5A were created using only 1. Figure 4 used a sample size of 50 to minimize error of the mean. |
