# Supplementary material for: A simulation of the random and directed motion of dendritic cells in chemokine fields
Source: PLoS Comput Biol. 2019 Oct 7;15(10):e1007295. doi: 10.1371/journal.pcbi.1007295 (PMC6797211; doi:10.1371/journal.pcbi.1007295)
Supplement: S1 Text — (DOCX) [file pcbi.1007295.s010.docx]

**Supplemental Information**

**1. Differences between Chan and Odde model and the current model**

The majority of the differences between the Chan and Odde model have already been accounted for in the body of the paper. However, while we have primarily used the same parameter values as they have, we have changed three parameters: We have reduced the number of clutches (40 vs. 75), increased the number of myosin motors (120 vs. 75), and adjusted the molecular clutch spring constant to 0.01pN/nm, as compared to the 5pN/nm value used by Chan and Odde. This is still consistent with experiment [1].

We have also been able to recreate Chan and Odde’s “substrate position” graph (see Science 80, 322, 2008, Figure 1C [2]). Our recreation is shown in Figure S1.

**2. Description of Chan and Odde’s Model** [2]

Chan and Odde modeled filopodia stochastically as a single F-actin filament undergoing retrograde flow as the result of a set of n_m_ myosin motors. This filament is assumed to be relatively noncompliant relative to the substrate and molecular clutches. A set of n_c_ clutches reversibly binds the filament. Once bound, these clutches can be deformed, and are modeled as linear, Hookean springs that fail only at the same location they bind the filament. When deformed, the clutches exert a spring force on the substrate beneath the cell, which is also modeled as a Hookean spring. Every time step, the position of the substrate is given by a simple force balance between the clutches and the substrate. As the substrate deforms, it exerts a force against the myosin motors, thus slowing their retrograde flow velocity in accordance with a linear force-velocity relationship.

**3. Parameters**

The model has several parameters. The following tables cover all of them in conventional units. When used to simulate behavior, values were converted to μg, μm, s-based units, with concentrations measured in molecules per cubic micron. This was done to ensure all numerical values were close to the order of unity. All parameters are available through S1-S4 Table.

**3. a. Filopodial Parameters**

Note that all filopodial parameters were kept constant throughout all simulations.

**3. b. Cellular Parameters**

Note that all cellular parameters were kept constant throughout all simulations.

**3. c. Gradient Parameters**

In contrast to the filopodial and cellular parameters listed above, many of the gradient parameters were given several values throughout the course of this study, primarily in the creation of Figures 3, 4, and especially 5. Almost all variables were set to zero for chemokinetic simulations, and given nonzero values for chemotactic simulations. K_d_ values were completely constant. Each value is marked accordingly.

Values have also indicated the average concentration of the chemokine over the region as a function of the K_d_. Finally, note that when used in simulation, concentrations were expressed in molecules per cubic micron, because this brings most quantities close to the order of unity.

**4. Supplemental Videos**

Supplemental videos 1 and 2 are both schematic representations of DC chemotaxis and eventually reaching a line of equistimulation. Red filopodia are exerting more force on the cell body, while blue filopodia are exerting less force. One frame represents 10 seconds of motion (2000 simulation ticks).

Supplemental videos 3 and 4 are both schematic representations of DC chemokinesis; red filopodia are exerting more force on the cell body, while blue filopodia are exerting less force. One frame represents 10 seconds of motion (2000 simulation ticks).

**5. Phase Behavior of Line of Equistimulation**

Of particular importance when considering the behavior of cells in countergradients are combinations of relative strength and slope that lead to no net directional motion (i.e. a line of equistimulation at x=0). This is important primarily because cells clustering at x=0 indicates that the two gradients are equal in strength. Using the data from Figure 6, we have approximated a smooth curve that shows all combinations of ε_1_ and α_2_ that should lead to approximately no directional motion, and thus which lead to equally effective gradients (see Figure S2).

**References**

1. Li F, Redick SD, Erickson HP, Moy VT. Force measurements of the alpha5beta1 integrin-fibronectin interaction. Biophys J. 84:1252-1262 (2003);

2. Chan CE, Odde DJ. Traction dynamics of filopodia on compliant substrates. Science 322(5908): 1687-1691 (2008).

3. Lele TP, Thodeti CK, Pendse J, Ingber DE. Investigating complexity of protein-protein interactions in focal adhesions. Biochem Biophys Res Commun. 369(3): 929-934 (2008);

4. Jiang G, Giannone G, Critchley DR, Fukumoto E, Sheet MP. Two-piconewton slip bond between fibronectin and the cytoskeleton depends on talin. Nature 424(6946):334-337 (2003).

5. Molloy JE, Burns JE, Kendrick-Jones B, Tregear RT, White DCS. Movement and force produced by a single myosin head. Nature 378(6553):209-212 (1995).

6. Ricart BG, John B, Lee D, Hunter CA, Hammer DA. Dendritic Cells Distinguish Individual Chemokine Signals through CCR7 and CXCR4. J Immunol 186(1):53-61 (2011).

7. Patla I, Volberg T, Elad N, Hirschfeld-Warneken V, Grashoff C, Fässler R, et al. Dissecting the molecular architecture of integrin adhesion sites by cryo-electron tomography. Nat Cell Biol. 12(9): 909-915 (2011);
